# Gray Matter Atrophy Is Primarily Related to Demyelination of Lesions in Multiple Sclerosis: A Diffusion Tensor Imaging MRI Study

**DOI:** 10.3389/fnana.2017.00023

**Published:** 2017-03-29

**Authors:** Eszter Tóth, Nikoletta Szabó, Gergõ Csete, András Király, Péter Faragó, Tamás Spisák, Krisztina Bencsik, László Vécsei, Zsigmond T. Kincses

**Affiliations:** ^1^Department of Neurology, Albert Szent-Györgyi Clinical Centre, University of SzegedSzeged, Hungary; ^2^Central European Institute of Technology, Masaryk UniversityBrno, Czechia; ^3^Department of Nuclear Medicine, University of DebrecenDebrecen, Hungary; ^4^Neuroscience Research Group of the Hungarian Academy of Sciences and University of SzegedSzeged, Hungary

**Keywords:** brain atrophy, demyelination, normal-appearing white matter, periventricular white matter, multiple sclerosis

## Abstract

**Objective:** Cortical pathology, periventricular demyelination, and lesion formation in multiple sclerosis (MS) are related (Hypothesis 1). Factors in the cerebrospinal fluid close to these compartments could possibly drive the parallel processes. Alternatively, the cortical atrophy could be caused by remote axonal transection (Hypothesis 2). Since MRI can differentiate between demyelination and axon loss, we used this imaging modality to investigate the correlation between the pattern of diffusion parameter changes in the periventricular- and deep white matter and the gray matter atrophy.

**Methods:** High-resolution T1-weighted, FLAIR, and diffusion MRI images were acquired in 52 RRMS patients and 50 healthy, age-matched controls. We used EDSS to estimate the clinical disability. We used Tract Based Spatial Statistics to compare diffusion parameters (fractional anisotropy, mean, axial, and radial diffusivity) between groups. We evaluated global brain, white, and gray matter atrophy with SIENAX. Averaged, standard diffusion parameters were calculated in four compartment: periventricular lesioned and normal appearing white matter, non-periventricular lesioned and normal appearing white matter. PLS regression was used to identify which diffusion parameter and in which compartment best predicts the brain atrophy and clinical disability.

**Results:** In our diffusion tensor imaging study compared to controls we found extensive alterations of fractional anisotropy, mean and radial diffusivity and smaller changes of axial diffusivity (maximal *p* > 0.0002) in patients that suggested demyelination in the lesioned and in the normal appearing white matter. We found significant reduction in total brain, total white, and gray matter (patients: 718.764 ± 14.968, 323.237 ± 7.246, 395.527 ± 8.050 cm^3^, controls: 791.772 ± 22.692, 355.350 ± 10.929, 436.422 ± 12.011 cm^3^; mean ± SE), (*p* < 0.015; *p* < 0.0001; *p* < 0.009; respectively) of patients compared to controls. The PLS analysis revealed a combination of demyelination-like diffusion parameters (higher mean and radial diffusivity in patients) in the lesions and in the non-lesioned periventricular white matter, which best predicted the gray matter atrophy (*p* < 0.001). Similarly, EDSS was best predicted by the radial diffusivity of the lesions and the non-lesioned periventricular white matter, but axial diffusivity of the periventricular lesions also contributed significantly (*p* < 0.0001).

**Interpretation:** Our investigation showed that gray matter atrophy and white matter demyelination are related in MS but white matter axonal loss does not significantly contribute to the gray matter pathology.

## Introduction

Multiple sclerosis is an inflammatory, demyelinating, neurodegenerative disease of the central nervous system. Besides the white matter lesions, which are the diagnostic cornerstones of the disease, increasing attention is being paid to gray matter atrophy, which has recently become a tool for the follow-up of the therapeutic efficacy (Kincses et al., 2014; De Stefano et al., 2016). The importance of the gray matter atrophy lies in the high correlation with the clinico-cognitive functioning (Roosendaal et al., 2011; Batista et al., 2012). However, the exact mechanism of the atrophy is not well-understood yet.

The demyelination in the gray matter is comparable in extent to that in the white matter (Gilmore et al., 2009). *Ex-vivo* studies have shown that the demyelination is mainly subpial (Type III lesions) and presents in the form of ribbons, often affecting several adjacent gyri. This kind of cortical demyelination proved to be associated with meningeal inflammation. Earlier studies found that a non-targeted general immunopathological response arising from this meningeal inflammation and mediated by the cerebrospinal fluid is responsible for the cortical pathology (Magliozzi et al., 2010; Lisak et al., 2012).

Along these lines, Jehna et al. reported a correlation between the increased periventricular lesion burden and the cortical atrophy in multiple sclerosis patients, which strongly supports the concept of a common cerebrospinal fluid-mediated pathology in the cortex and in the periventricular white matter (Jehna et al., 2015).

In contrast, leukocortical lesions (Type I) are almost as abundant as subpial lesions (Wegner et al., 2006), and neuronal loss was detected in regions where no B-cell follicle-like structures were detected. These findings suggest that other mechanisms are also involved in the cortical atrophy. Remote axonal transections (putatively related to lesion formation in the white matter and demyelination) have also been suggested to account for the cortical atrophy. The dying-back axonopathy could ultimately result in atrophy of the cortical gray matter (Trapp and Nave, 2008; Geurts et al., 2012). The reduced cortical input might also lead to plastic changes, such as a reduction in synaptic density, which might also present in the form of cortical atrophy. This hypothesis is strengthened by the findings that most significant cortical atrophy in multiple sclerosis is in association cortices such as the cingulate cortex, which has extensive cortico-cortical connections (Charil et al., 2007).

Magnetic resonance spectroscopy studies demonstrated a low level of N-acetyaspartate—a marker of neuronal viability—in the normal appearing white matter of multiple sclerosis patients, indicating loss or dysfunctional axons (Fu et al., 1998; Wood et al., 2012). It was shown that the N-acetyaspartate/myo-inositol ratio—a putative marker of reduced neuronal integrity and increased gliosis—significantly contributes to brain volume change (Llufriu et al., 2014). The myelin pathology in the normal appearing white matter as measured by magnetization transfer ratio was also correlated to the cerebral atrophy and disability (Vrenken et al., 2007).

Based on the above mentioned results we aimed to investigate the contribution of the white matter pathology to the cortical atrophy in multiple sclerosis. Firstly, we explored if the focal lesional pathology or the more diffuse pathology of the normal appearing white matter contributes more to the gray matter atrophy. Secondly, based on Jehna's results (Jehna et al., 2015), we investigated if the periventricular white matter has a special role in the development of gray matter atrophy.

We used diffusion tensor imaging to investigate the white matter pathology. Diffusion tensor imaging describes the diffusion of water in biological tissues non-invasively. The molecular diffusion is hindered by cellular elements (mainly membranes), and hence the diffusion profile of the water can reveal microscopic details about the tissue architecture. Importantly, axon loss and demyelination cause different diffusion profile alterations. The changes in axial diffusivity relates to axon damage, while the alterations of radial diffusivity refer to myelin damage, which in our study we will allude to as demyelination-like diffusion parameters.

Gray matter atrophy defined by demyelination-like diffusion features would support the common origin of white matter demyelination and gray matter atrophy (possibly a common cerebrospinal fluid-mediated pathology of the gray matter and the periventricular white matter; Hypothesis 1). Gray matter atrophy related to an axon loss-like diffusion pattern would suggest the causative factor of remote axonal transection in gray matter atrophy (Hypothesis 2).

The contribution of pattern of diffusion parameters to the gray matter atrophy was investigated by model-free partial least square approach. When the predictors are highly collinear, as it is expected in case of diffusion parameters, the use of conventional regression analysis is not recommended. Partial least squares not only deals with the issue of collinearity, but offers to identify a pattern of parameters that best predicts the variable to be explained.

## Materials and methods

### Subjects

The study was conducted on 52 patients with a diagnosis of relapsing remitting multiple sclerosis and 50 healthy, age-matched volunteer controls with no history of any neurological or psychiatric diseases. Patients were recruited from the Multiple Sclerosis Outpatient Clinic at the Department of Neurology. The diagnosis was based on the 2005 revision of the McDonald criteria (Polman et al., 2005). The clinical disability of the patients, as measured on the Kurtzke expanded disability status scale (EDSS; Kurtzke, 1983), was 1.66 ± 1.44. All patients were on disease-modifying therapy (Table 1). All patients were in a stable clinical condition, no relapses and no EDSS progression had occurred in the preceding 6 months.

**Table 1:** **Demographic and clinical data on the participating subjects**.

	**Patients**	**Controls**
*n*	52	50
Age (years; mean ± *SD*)	40.87 ± 10.31	37.14 ± 10.77
Sex (male)	13	20
Disease duration (years; mean ± *SD*)	9.69 ± 7.188	N.A.
EDSS score	1.66 ± 1.44	N.A.
Therapy	Interferon beta: 25, Glatiramer acetate: 16, Fingolimod: 1	

The study was approved by the ethics committee of the Medical University of Szeged and all study participants gave their written informed consent in accordance with the Declaration of Helsinki (Ref. No. 56/2011).

### Image acquisition

MR imaging were carried out on a 1.5T GE Signa Excite HDxt MR scanner. 3D spoiled gradient echo (FSPGR: TE: 4.1 ms, TR: 10.276 ms, matrix: 256 × 256, FOV: 25 × 25 cm, Flip angle: 15°, in-plane resolution: 1 × 1 mm, slice thickness: 1 mm), FLAIR (TE: 4.1 ms, TR: 10.276 ms, matrix: 256 × 256, FOV: 25 × 25 cm, Flip angle: 15°, in-plane resolution: 1 × 1 mm, slice thickness: 1 mm) and 60 direction diffusion-weighted images with 6 non-diffusion-weighted reference volumes (TE: 93.8 ms, TR: 16000 ms, matrix: 96 × 96, FOV: 23 × 23 cm, Flip angle: 90°, in-plane resolution: 2.4 × 2.4 mm slice thickness: 2.4 mm, b: 1000 s/mm^2^, NEX: 2, ASSET) were acquired for all subjects.

### Lesion distribution

Manual lesion segmentation was carried out on the FLAIR images by the first author and supervised by ZTK, who has substantial experience in multiple sclerosis neuroradiology. FLAIR images were registered to the high-resolution T1 images with 6 degree-of-freedom linear registration (Jenkinson et al., 2002). High-resolution T1 images were registered to standard space images with 12 degree-of-freedom affine registration and were refined by non-linear registration implemented in FNIRT (Andersson et al., 2007). Binary lesion masks were transformed to standard space by using the transformation matrices and warp fields from the above-mentioned registrations. Standard space masks were thresholded at 0.5 and binarized again in order to avoid the size increment of the mask caused by the trilinear interpolation. Binary masks were summed in order to provide a lesion probability distribution.

### Diffusion tensor analysis

Diffusion data were corrected for Eddy currents and movement artifacts by 12 degree-of-freedom affine linear registration to the first non-diffusion-weighted reference image. Diffusion images were processed by using FDT (FMRIB's Diffusion Toolbox part of FSL: http://www.fmrib.ox.ac.uk/fsl/fdt/). Fractional anisotropy, mean diffusivity [(λ_1_ + λ_2_ + λ_3_)/3), axial diffusivity (λ_1_) and radial diffusivity ((λ_2_ + λ_3_)/2] to the principal diffusion direction were computed for the whole brain.

We used the Tract-Based Spatial Statistics (TBSS) method to reduce possible errors resulting from misalignment of the images: A non-linear registration tool (FNIRT), which uses a b-spline representation of the registration warp field, aligned all fractional anisotropy images to a 1 × 1 × 1 mm FMRIB58_FA standard space. We brought the data on all patients into the standard space, and created the mean fractional anisotropy image, which was then fed into the fractional anisotropy skeletonization program, thresholded at fractional anisotropy 0.2 to create a mean fractional anisotropy skeleton that represented the centers of all tracts common to the group. The aligned fractional anisotropy data on each subject was then projected onto this skeleton, which resulted in the 4D skeletonized fractional anisotropy image. The resulting data was fed into voxel-wise cross-subject statistics.

With the use of a non-parametric permutation-based cluster analysis (5,000 permutation) as implemented in FSL, modeling and inferring, we accomplished the standard general linear model (GLM) design. The design encoded for group membership. Statistical tresholding was carried out with Threshold Free Cluster Enhancing (TFCE) approach (*p* < 0.05 was chosen as threshold and the results are corrected for multiple comparisons across space).

### Evaluation of global atrophy

We calculated the total brain volume with SIENAX (Smith et al., 2002), part of FSL (Smith et al., 2004; Jenkinson et al., 2012). SIENAX started by extracting brain and skull images from the single whole-head input data (Smith et al., 2002). We then carried out tissue-type segmentation with partial volume estimation (Zhang et al., 2001) in order to calculate the total volume of brain tissue (including separate estimates of volumes of total gray matter, peripheral gray matter and white matter). The area under the binary lesion masks were “filled” with intensities that are similar to those in the non-lesioned neighborhood, to reduce the bias caused by the T1 hypointense lesions (Battaglini et al., 2012).

We performed volumetric comparison across groups and the correlation tests with the Statistical Package for Social Sciences (SPSS 17 for OS X, SPSS Inc., http://www.spss.com).

### Relationship of brain atrophy and the compartmental diffusion metrics

A voxel-wise alteration of the diffusion parameters was calculated for each patient, by comparing the value of every voxel with the distribution from the normal subjects in the spatially matching voxel (z-score). To identify a global white matter damage we calculate the averages of these z-scores:

$$\begin{array}{l} {\overset{-}{X}}_{n}=\frac{\overset{q}{\underset{i=1}{\sum }}{X_{n,}}_{i}}{q},\\ \delta_{n}=\frac{\overset{q}{\underset{i=1}{\sum }}\left({\overset{-}{X}}_{n}-{X_{n,}}_{i}\right)}{q},\\ Z_{X,n,j}=\frac{X_{n,j}-X_{n}}{\delta_{n}}, \end{array}$$

where *X* is the measured diffusion parameter (fractional anisotropy, mean diffusivity, axial diffusivity, and radial diffusivity) in the *n*^*th*^ voxel in the skeleton. Indices *i* and *j* are for controls and patients, respectively.

The average diffusion parameters were calculated for each patient in the following compartments: periventricular lesions, non-periventricular lesions, periventricular normal white matter, and non-periventricular normal white matter.

To define the periventricular space the ventricles were manually segmented on the 1 × 1 × 1 mm FMRIB58_FA image dilated by three voxels similar to Jehna et al. (2015). The non-periventricular white matter was defined as the rest of the white matter, periventricular part excluded.

The lesions were projected to the fractional anisotropy skeleton. The manually segmented lesions were brought to the diffusion data space with 6 degree-of-freedom linear registration. Through use of the warp field and the skeleton projections of the TBSS analysis of the fractional anisotropy images, the lesion mask was brought to the skeleton with the FSL *tbss_non_fa* algorithm. The mask was finally thresholded at 0.5 and binarized to avoid any size increment arising from the interpolation.

We used Partial least square regression analysis to estimate the contributions of the calculated compartmental diffusion parameters to the EDSS, gray matter and global brain atrophy. If *Y* is an *n* × *q* matrix of dependent variables over *n* observations and *X* is an *n* × *p* matrix of predictors, Partial least squares successively extracts latent variables (factors and loadings) from *X* and *Y* in such a way that covariance between the factors and loadings is maximized. With this approach, Partial least squares reduces the dimensionality of the data by providing a weighted linear combination of *X* variables to form orthogonal components that predicts the dependent variable. In mathematical terms, Partial least squares is a linear decomposition of *X* and *Y* such that

$$\begin{array}{l} X=TP^{T}+E,\\ Y=UQ^{T}+F \end{array}$$

and the covariance between *T* and *U* is maximum (Abdi and Williams, 2013). In the above equations, *T* is the *n* × *r X* scores, *U* is the *n* × *r Y* scores, *P* is the *p* × *r X* loadings, *Q* is the *1* × *r Y* loadings, *E* and *F* are residuals, and *r* is the number of extracted latent variables. The statistical inference on the significance of the latent variable was carried out by permutation tests on the singular values of the decomposition. The elements of the dependent variable matrix were randomly permuted 5,000 times and the singular value was recalculated to depict a null distribution. The summary of the importance for the *X* loadings was calculated by a Variable Importance in the Projection score (Wold et al., 1994). Since the average of squared Variable Importance in the Projection scores is equal to 1, the “>1” rule was used for the selection of the important variables.

In the current setting, the dependent variable (*Y*) is the normalized brain or gray matter volume or EDSS, the predictors (*X*) are the diffusion parameters in the different compartments (periventricular and non-periventricular $\bar{Z}{\;}_{FA},\bar{Z}{\;}_{MD},\bar{Z}{\;}_{AD},\text{ and }\bar{Z}{\;}_{RD}$), and the observations are the patients. The *X* loadings are the optimum weights of the compartmental diffusion parameters which best predict the peripheral gray matter atrophy or EDSS.

## Results

### Lesion probability distribution

The average native space lesion load was 12.328 ± 16.100 cm^3^ (mean ± *SD*) and the lesion load normalized to the intracranial volume (v scaling factor) was 17.087 ± 22.509 cm^3^ (mean ± *SD*). The normalized lesion volume showed a negative correlation with the normalized gray matter volume (*R* = −0.32, *p* < 0.021), but no correlation was found with the normalized brain volume. The lesion load did not correlate with the EDSS of the patients either.

**Figure 1:**
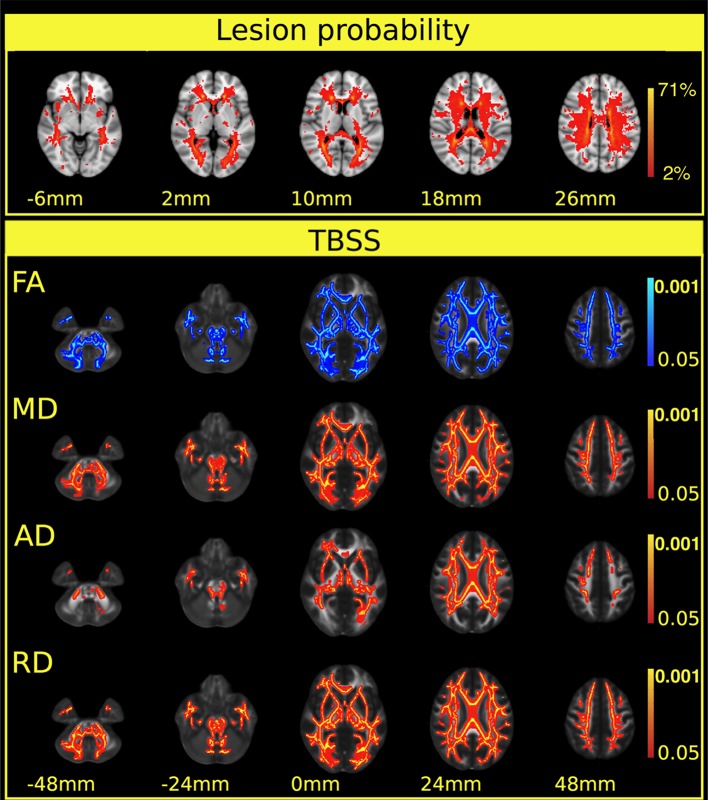
**Extensive diffusion parameter changes and cortical atrophy were found in multiple sclerosis patients**. The first row depicts the lesion probability map of the 52 patients. The colorbar represents the number of patients having a lesion in a particular localization. The lesion probability map was overlaid on the MNI152 standard brain. The z coordinates of the selected slices are shown below the images. The next section of the Figure depicts the results of the TBSS analysis. Significant differences in FA, MD, and axial and radial diffusivity between patients and controls in the white matter skeleton are shown in the consecutive rows. A blue color indicates decrease, and red-to-yellow colors an increase in the given diffusion parameters. A thickened version of the significant cluster is used for easier visualization (red-to-yellow or blue shades). Colorbars represent *p*-values (corrected for multiple correlation). Statistical images are overlaid on the FMRIB58_FA standard FA template and the z coordinates are shown below the images. The *z*-values under the significant corrected *p*-values (*p* < 0.05) are shown. Images are overlaid on the MNI152 standard brain and the z coordinates are shown under the images.

The lesions were distributed across widespread white matter regions, but the lesion probability was highest in the periventricular white matter (Figure 1 first row).

### Atrophy and diffusion alterations in multiple sclerosis

**Figure 2:**
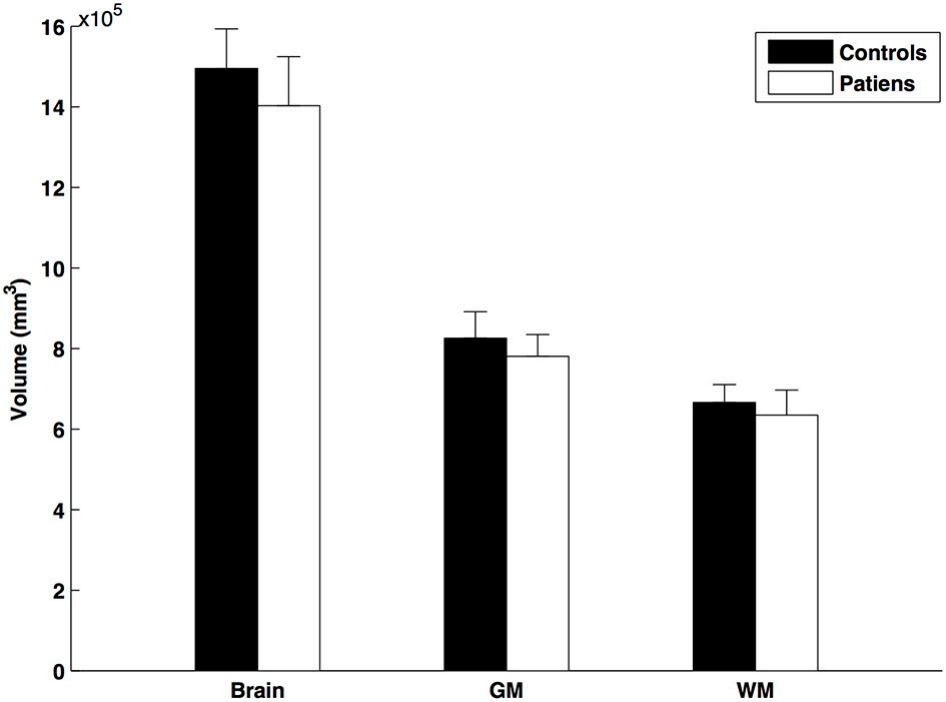
**Normalized brain volumes of patients and controls**. The error bars represent the standard errors.

The SIENAX analysis revealed reduction in total brain volume (patients: 718.764 ± 14.968 cm^3^, controls: 791.772 ± 22.692; mean ± SE), total white matter (patients: 323.237 ± 7.246 cm^3^, controls: 355.350 ± 10.929; mean ± SE), and total gray matter (patients: 395.527 ± 8.050 cm3, controls: 436.422 ± 12.011; mean ± SE) volume in the multiple sclerosis patients compared to controls (normalized to the premorbid brain volume, the normality of the data was violated in the Kolmogorov-Smirnov test, and hence the non-parametric Mann-Whitney *U*-test was used: *p* < 0.015; *p* < 0.0001; and *p* < 0.009, respectively; Figure 2).

Relative to the healthy controls, TBSS indicated significant reductions of fractional anisotropy (*p* < 0.0002) in the multiple sclerosis patients: in essentially all the white matter fiber bundles, with the exception of the area of the corticospinal tract (Figure 1 second row). The mean diffusivity was increased (*p* < 0.0002) in the majority of the white matter bundles but not in the corticospinal tracts and the inferior longitudinal fasciculus (Figure 1 third row, Table 2). The radial diffusivity also displayed a significant increase (*p* < 0.0002) in all examined fibers in the skeleton, apart from the corticospinal tracts (Figure 1 fourth row, Table 2). Importantly, the white matter, diffusion alterations (fractional anisotropy, mean diffusivity, and radial diffusivity) were distributed in the normal-appearing white matter and also in the periventricular white matter where lesions appeared with high probability.

**Table 2:** **The local maxima of significant group differences for AD, RD, and FA as fund by FSL's ***cluster*** algorithm**.

	**Anatomy**	**x**	**y**	**z**
AD	Right Inferior fronto-occipital fasciculus	26	23	14
	Left Inferior fronto-occipital fasciculus L	−25	25	14
	Forceps minor	−10	26	14
	Forceps major	−25	−72	15
	Forceps major	−26	−67	15
	Forceps major	−29	−62	15
RD	Forceps minor	21	21	34
	Right inferior fronto-occipital fasciculus	30	37	−1
	Forceps minor	−8	36	−1
	Right Inferior fronto-occipital fasciculus	32	11	−1
	Left Inferior front-occipital fasciculus	−32	8	−1
	Right superior longitudinal fasciculus	34	−46	21
MD	Forceps minor	−10	33	2
	Right superior longitudinal fasciculus	34	2	32
	Right Inferior fronto-occipital fasciculus	32	8	2
	Right superior longitudinal fasciculus	34	−3	2
	Left superior longitudinal fasciculus	−30	3	32
	Left Cingulum	−20	−45	2
FA	Right inferior fronto-occipital fasciculus	27	38	−2
	Right inferior fronto-occipital fasciculus	28	40	−2
	Forceps minor	20	45	−2
	Left inferior fronto-occipital fasciculus	−28	−82	−1
	Left inferior fronto-occipital fasciculus	−31	−70	−1
	Right Cingulum	22	−60	−1

*x, y, and z coordinates are in MNI space. p < 0.0001 in all cases. Anatomical localizations are given according to the John Hopkins University white−matter tractography atlas as implemented in fslview (Hua et al., 2008)*.

In contrast, an increase in axial diffusivity was found only in the more central fibers and in the intrathalamic white matter (*p* < 0.0002) in the corpus callosum, the superior longitudinal fasciculus, the superior corona radiate, the inferior fronto-occipital fasciculus, the posterior and anterior thalamic radiation, and the internal capsule (Figure 1 fifth row, Table 2).

### The relationship of brain atrophy and the compartmental white matter pathology

**Figure 3:**
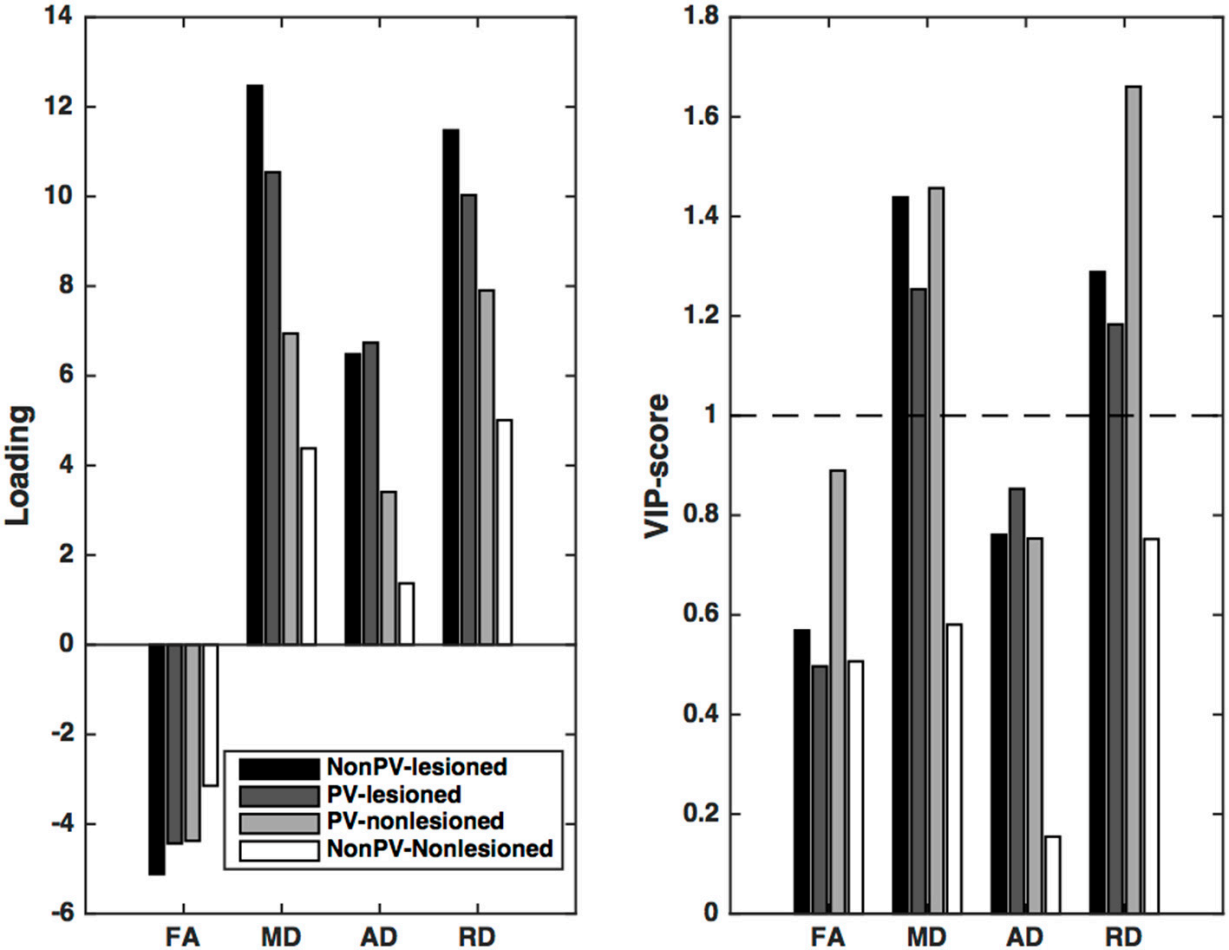
**Partial least squares loadings and VIP scores that describe the optimum contrast of the independent variables that predict the gray matter volume**. From these loadings and VIP scores, it is conceivable the predominantly the diffusion parameters of the lesions and the non-lesioned periventricular white matter (PV-WM) drive the gray matter atrophy. Of the diffusion parameters, MD and RD, related most significantly to the gray matter atrophy. VIP scores are considered significant if higher than 1.

In the first Partial least squares analysis the normalized gray matter volume was used as dependent variable. Only the first latent variable was evaluated, because the second latent variable explained only a small fraction of the variance of the dependent measure (<5%) and the permutation test revealed a non-significant latent variable. The permutation test indicated that the first latent variable was significant (*p* < 0.001) and accounted for 47.3% of the variation of the dependent variable and 76.5% of the predictors. The *X* loadings and the corresponding Variable Importance in the Projection scores indicated that the mean diffusivity and radial diffusivity of the lesioned and non-lesioned periventricular and the non-periventricular lesioned white matter contributed significantly to the gray matter atrophy (Figure 3, Table 3).

**Table 3:** **The loadings and VIP scores predicting the normalised brain volume**.

**Diffusion parameter**	**Compartment**	**Loadings**	**VIP-scores**
FA	Non-PV Lesioned	5.11	0.56
	PV Lesioned	4.43	0.49
	PV Non-lesioned	4.37	0.88
	Non-PV Non-lesioned	3.14	0.50
MD	Non-PV Lesioned	−12.46	1.43^*^
	PV Lesioned	−10.54	1.25^*^
	PV Non-lesioned	−6.94	1.45^*^
	Non-PV Non-lesioned	−4.38	0.58
AD	Non-PV Lesioned	−6.48	0.76
	PV Lesioned	−6.73	0.85
	PV Non-lesioned	−3.40	0.75
	Non-PV Non-lesioned	−1.37	0.15
RD	Non-PV Lesioned	−11.47	1.28^*^
	PV Lesioned	−10.03	1.18^*^
	PV Non-lesioned	−7.90	1.66^*^
	Non-PV Non-lesioned	−5.01	0.75

* *VIP scores higher than 1 were considered to indicate a significant contribution*.

Similar results were found in the case of the normalized brain volume: only the first latent variable was significant according to the permutation test. The first latent variable accounted for 24.9% of the variation of the dependent variable and 76.7% of the predictors. *X* loadings coding the optimum contrast of the predictors exhibited a similar pattern as for the gray matter volume.

**Figure 4:**
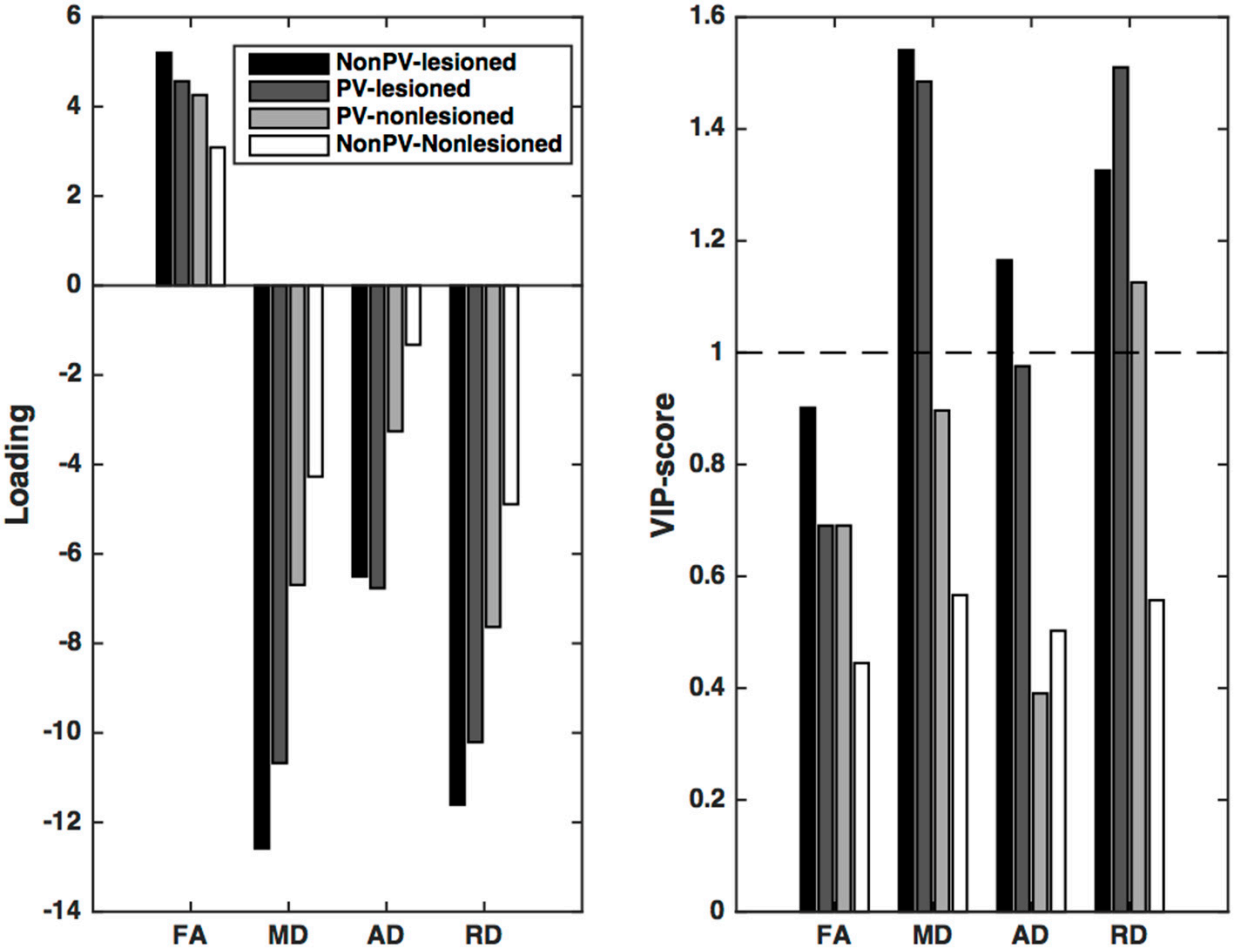
**Partial least squares loadings and VIP scores that describe the optimum contrast of the independent variables that predict the disability (EDSS) of the patients**. EDSS can be predicted most significantly from the RD and MD of the lesions and the periventricular non-lesioned white matter. A further contribution can be seen from the AD change in the periventricular lesions. VIP scores are considered significant if higher than 1.

In an analysis in which the EDSS was the dependent variable, the first latent variable was significant according to the permutation test (*p* < 0.001, explained variance of the independent variable: 18.8%, and of the predictors: 76.8%). The *X* loadings showed that the predictors of EDSS were very similar to those of the gray matter and the brain volume: the radial diffusivity and the mean diffusivity of the lesions had the largest effects on EDSS, irrespective of the distance from the ventricles. Furthermore, the radial diffusivity of the non-lesioned periventricular white matter and the axial diffusivity of the periventricular lesions contributed significantly (Figure 4).

## Discussion

In this diffusion tensor imaging study, we investigated the relationship between the gray matter atrophy and the microstructure of the white matter in four compartments in relapsing-remitting multiple sclerosis. We hypothesized that, if the gray matter atrophy was related to axon-loss-like diffusion pattern alterations, that would be an indication of secondary cortical atrophy due to remote axonal transection. Alternatively, if the cortical atrophy correlated with a demyelination-like diffusion pattern, a common pathomechanism could be suspected. Furthermore, if predominantly the periventricular demyelination correlated with the atrophy, common cerebrospinal fluid mediated processes could be suspected. Our model-free, partial least squares analysis supported the second hypothesis, i.e., the gray matter atrophy was related predominantly to the increased mean diffusivity and radial diffusivity of the lesions and the periventricular non-lesioned white matter, a pattern of diffusion parameters that is a putative signature of demyelination. A similar pattern of relationship was found with the disability of the patients. In both cases, the alteration in axial diffusivity, a putative marker of axon loss, contributed less. Importantly, the pathology of the normal-appearing white matter did not contribute significantly to the atrophy.

Previous studies have demonstrated that the different patterns of the diffusion parameter alterations may indicate various pathological white matter changes. In mouse models of multiple sclerosis (Song et al., 2005; Velicu et al., 2012), the changes observed in axial diffusivity and radial diffusivity were suggested to relate to axon or myelin damage, respectively. A mouse model study revealed a decreased fractional anisotropy in transected nerves, with the fractional anisotropy returning toward normal with axonal regeneration. Moreover, fractional anisotropy and axial diffusivity correlated significantly with the total number of axons (Lehmann et al., 2010). Three days after induction of ischaemia in the retina, a significant decrease in axial diffusivity was observed in mice without any detectable changes in radial diffusivity, which was consistent with the histological findings of significant axonal degeneration without demyelination. Two days later, consistently with the histological finding of myelin degeneration, the radial diffusivity was increased (Song et al., 2003). Work with a novel mouse model, that combined cuprizone-induced demyelination and experimental autoimmune encephalomyelitis indicated that axonal damage and cellular infiltration led to an alteration in axial diffusivity, whereas primary demyelination after cuprizone treatment was reflected by changes in radial diffusivity, but not in axial diffusivity (Boretius et al., 2012). Moreover, the myelin content in the postmortem human brain prior to and after fixation was predicted by the changes in radial diffusivity, fractional anisotropy and mean diffusivity (Schmierer et al., 2008).

According to the results described above, the diffusion parameter changes found in multiple sclerosis patients reflect widespread demyelination in the white matter. These results are similar to others presented earlier (Cifelli et al., 2002; Fabiano et al., 2003; Inglese et al., 2004; Poonawalla et al., 2008; Dineen et al., 2009; Roosendaal et al., 2009; Raz et al., 2010; Kern et al., 2011; Yu et al., 2012). However it is important to point out that we also found a signature of extensive demyelination in the normal appearing white matter, which was only rarely described in former diffusion tensor imaging studies. The high number of diffusion directions used in our study most probably increased the sensitivity of our investigation. A possible histopathological correlate of this change in mean diffusivity might be the lipid abnormality found in the diffusely abnormal white matter (Laule et al., 2013).

More importantly, a pattern of diffusion parameter changes resembling demyelination in the lesions and in the non-lesioned periventricular white matter was a strong predictor of gray matter atrophy. Furthermore, the microstructure of the non-periventricular normal-appearing white matter, even if it had significant demyelination, contributed less to the gray matter pathology. The relatively small contribution of axial diffusivity alterations to the gray matter atrophy, disprove our first hypothesis, that the cortical atrophy is secondary to axonal damage in the white matter. Alternatively, the diffusion measures have only a low sensitivity for axonal damage.

More interestingly, the demyelination-like pattern of diffusion parameters in the non-lesioned periventricular white matter strengthens the hypothesis of Jehna (Jehna et al., 2015) that periventricular demyelination and cortical atrophy are driven by the same process, most probably arising from the nearby Cerebrospinal fluid. Meningeal inflammation, B-cell follicle-like structures, CD3^+^, and CD8^+^ T-cell infiltrates (Magliozzi et al., 2010; Howell et al., 2011) were associated with subpial demyelination (Type III lesions) and cortical atrophy. Apart from the demyelination, a gradient of neuronal loss toward the pial surface was observed in the cortex, accompanied by astrocyte loss and, opposite to this gradient, microglia activation (Magliozzi et al., 2010). These findings are consistent with those of *in vitro* studies (Lisak et al., 2012) and suggest that a non-targeted general immunopathological response mediated by the B-cells and CD8^+^ T-cells via cytotoxic tissue damage or indirectly through the activation of microglia might be responsible for the cortical pathology. A common pathomechanism behind the cortical demyelination and periventricular lesion is further strengthened by the spatial location of the abnormalities. Subpial lesions are frequently present around deep sulci that often have expanded Virchow-Robin space, a cerebrospinal fluid space with abundant immune cells (Trapp and Nave, 2008). The periventricular white matter has a special feature in contrast with other deep white matter regions, that it is in close proximity to the cerebrospinal fluid. Similarly, the periventricular lesions are often formed around the venules and the Virchow-Robin spaces have been shown to be enlarged in multiple sclerosis (Ge et al., 2005; Wuerfel et al., 2008).

Since the clinical disability and gray matter atrophy are highly correlated, it was expected that the pattern of diffusion parameters best predicting the EDSS would be similar to that of those predicting the gray matter volume. However, one crucial difference must be noted: the axial diffusivity of the periventricular lesions, a putative sign of axon damage, also contributed significantly to the disability. This finding confirms earlier results indicating a significant correlation between N-acetyaspartate (a marker of neuronal/axonal integrity) and disability (Llufriu et al., 2014).

Our previous investigation also revealed that the pattern of various diffusion parameters can describe a pathology better than can the individual parameters (Kincses et al., 2013). The partial least squares analysis have a benefit over conventional multiple linear regression analyses as it can identify a pattern of predictors. It is especially useful in settings when the non-collinearity assumption is violated.

## Conclusions

In our study we found extensive alterations in diffusion parameters in the patients that suggested demyelination in the high lesion burden periventricular white matter and in the normal-appearing white matter. In the patients we found significant brain, gray and white matter atrophy. The partial least squares analysis revealed a combination of demyelination-like diffusion parameters, in the lesions and in the non-lesioned periventricular white matter which best predicted the gray matter atrophy. Similarly, EDSS was best predicted by the radial diffusivity of the lesions and the non-lesioned periventricular white matter. The periventricular lesion axial diffusivity also contributed significantly to the clinical disability.

**Figure 5:**
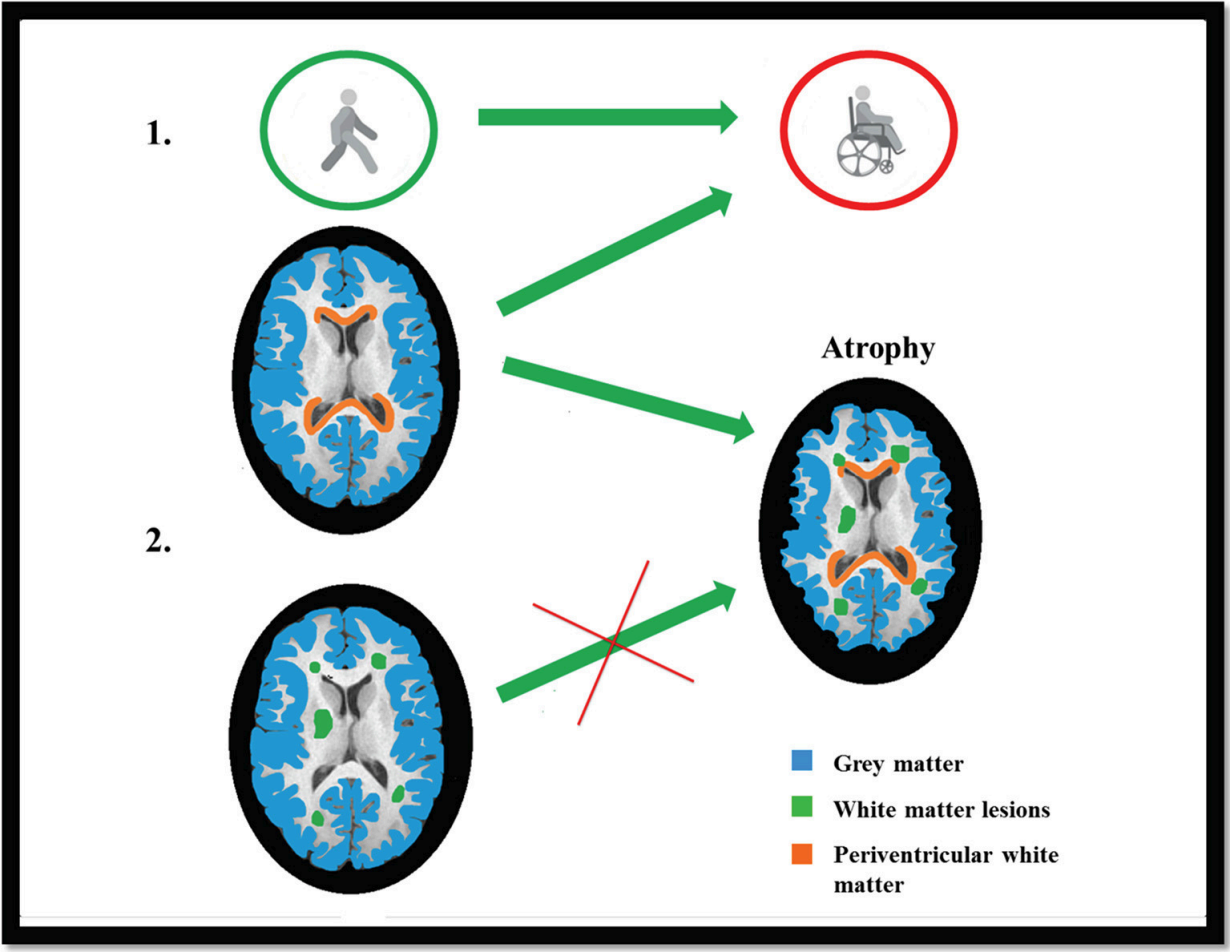
**Graphical Presentation: EDSS was best predicted by the radial diffusivity of the lesioned and non-lesioned periventricular white matter and also the axial diffusivity of the lesioned periventricular white matter (Part 1)**. The gray matter atrophy (marked with blue color) was best predicted by the combination of demyelination-like diffusion parameters (Hypothesis I.), in the lesions and in the non-lesioned periventricular white matter (marked with red color), but not by the axonloss-like diffusion parameters in the NAWM (Hypothesis II; lesions are marked with green color; Part 2).

The pathology of multiple sclerosis is a heterogeneous process, but our results demonstrate that even spatially remote processes may have common roots. The substance mediating between such processes might be the cerebrospinal fluid. Further studies are needed to reveal the factors that are responsible for the gray and white matter demyelination. We summarized our results in a Graphical presentation (Figure 5).

## Author contributions

The authors concur with the submission of the manuscript and both authors have approved the final version. Everyone who was working on the manuscript is listed between the authors. ET and ZK: Design of the work, interpretation of data for the work, Drafting the work, Final approval of the version to be published, and Agreement to be accountable for all aspects of the work in ensuring that questions related to the accuracy. NS: Interpretation of data for the work, Drafting the work, Final approval of the version to be published, and Agreement to be accountable for all aspects of the work in ensuring that questions related to the accuracy. GC, AK, PF, and TS: Analysis, Revising the work critically for important intellectual content, Final approval of the version to be published, and Agreement to be accountable for all aspects of the work in ensuring that questions related to the accuracy. KB and LV: Substantial contributions to the conception, drafting the work, Final approval of the version to be published, and Agreement to be accountable for all aspects of the work in ensuring that questions related to the accuracy.

### Conflict of interest statement

The authors declare that the research was conducted in the absence of any commercial or financial relationships that could be construed as a potential conflict of interest.
